# An Ostensive Information Architecture to Enhance Semantic Interoperability for Healthcare Information Systems

**DOI:** 10.1007/s10796-023-10379-5

**Published:** 2023-03-01

**Authors:** Hua Guo, Michael Scriney, Kecheng Liu

**Affiliations:** 1 School of Electronic Engineering and Computer Science, Queen Mary University of London, London, UK; 2 Insight Centre for Data Analytics, School of Computing, Dublin City University, Dublin, Ireland; 3 Informatics Research Centre, University of Reading, Reading, UK

**Keywords:** Healthcare information systems, Semantic interoperability, Digital healthcare, FHIR, Ostensive approach, Graph database, Ontology graph, Knowledge graph

## Abstract

Semantic interoperability establishes intercommunications and enables data sharing across disparate systems. In this study, we propose an ostensive information architecture for healthcare information systems to decrease ambiguity caused by using signs in different contexts for different purposes. The ostensive information architecture adopts a consensus-based approach initiated from the perspective of information systems re-design and can be applied to other domains where information exchange is required between heterogeneous systems. Driven by the issues in FHIR (Fast Health Interoperability Resources) implementation, an ostensive approach that supplements the current lexical approach in semantic exchange is proposed. A Semantic Engine with an FHIR knowledge graph as the core is constructed using Neo4j to provide semantic interpretation and examples. The MIMIC III (Medical Information Mart for Intensive Care) datasets and diabetes datasets have been employed to demonstrate the effectiveness of the proposed information architecture. We further discuss the benefits of the separation of semantic interpretation and data storage from the perspective of information system design, and the semantic reasoning towards patient-centric care underpinned by the Semantic Engine.

## Introduction

A health information system (HIS) manages healthcare data and supports decision-making in order to improve the quality of health services. The nature of demands, particularly resulting from patient-centred care policies (Stewart, 2001) and evidence-based medicine (Sackett, 1997), necessitates the efficient management and usage of healthcare resources. In view of the benefits provided to healthcare services by advances such as sensor-based technology and the ubiquitous computing environments for multiple HIS users, including physicians, patients, funders of healthcare, and regulatory bodies (He et al., 2019), the HIS faces multiple challenges (Haux, 2006). The HIS landscape has significantly expanded, with its complexity increasing exponentially; the examination of this fundamental issue in terms of HIS architecture, therefore, has great importance. In response to increased levels of connectivity and stakeholder demand, HISs are evolving into healthcare ecosystems; facilities of this nature should have the capacity to deal with multiple domains of knowledge (Blobel, 2019), particularly heterogeneous data collected by novel medical devices or sensors (Kankanhalli et al., 2016).

Interoperability facilitates intercommunication, enabling data sharing across disparate information systems (Geraci, 1990; Mouttham et al., 2012). The diversity of datasets generated by information obtained from wearable devices, telehealth, and digital therapeutics (Aungst & Patel, 2020; Li et al., 2015) requires exchangeability, not only of the data themselves but also of the information they contain. Accordingly, the issue of the interoperability of digital ecosystems is receiving an increasing amount of attention from both academia and industry (Grimson et al., 2000).

From information network connectivity to application interaction, interoperability can be categorised into three forms, specifically: technical, syntactic, and semantic (Joshi et al., 2017; Tolk et al., 2007). In the dimensions of technical and syntactic interoperability, consensus solutions have, to some extent, been developed; for example, information exchange protocols such as REST (Resource Representational State Transfer) API and unified data formats are, in practice, becoming more widely adopted. However, there remain significant challenges for semantic interoperability, which concerns the capacity of systems to interpret the meaning of the exchanged information within ecosystems. Because the applications of artificial intelligence, advanced data analytics, and wearable technologies are becoming increasingly common within healthcare ecosystems, the subject of interaction between heterogeneous applications and systems has attracted the interest of many academics. This research explores the meaningful information exchange between two or more entities within a healthcare ecosystem at the semantic level (Liu & Li, 2015; Ouksel & Sheth, 1999) from the perspective of information systems, proposing a new architecture for HIS designed to support the delivery of high-quality care.

In view of the complex nature of medical information representations, international standards have been produced in order to achieve their semantic interoperability, such as HL7 v2, and v3 (HL7 International, 1987), open EHR (open EHR, 2003) and CEN/ISO 13606 (ISO) (European Committee for Standardization (CEN), 2019). Although these standards claim to solve the problem of semantic exchange, from the perspective of information exchange, they are actually applied at different levels of information systems, i.e. syntactic, semantic, and pragmatic (Liu & Li, 2015). However, the semantic ambiguity persists (Dolin et al., 2018; Jiang et al., 2015, 2016).

Because of the sub-optimal performance of these standards, especially at semantic and pragmatic levels, all continue to evolve. FHIR (Fast Health Interoperability Resources) (HL7 International, 2011) is the latest version of HL7; it is applicable in the majority of healthcare scenarios and has been adopted by all UK hospitals, and those in many other countries. One of the main reasons for the healthcare sector’s wide acceptance of FHIR is its excellent compatibility with Internet protocols and its ease of deployment (Bender and Sartipi, 2013). However, with its widespread application in industry, FHIR’s limitations in terms of semantic interoperability create ambiguity (Jiang et al., 2015, 2016), leading researchers to address the issues associated with its implementation (Dolin et al., 2018; Jiang et al., 2015). Focusing on its limitations, this paper proposes an ostensive information architecture for the enhancement of FHIR’s interoperability in digital healthcare systems.

The ostensive approach, which defines concepts by direct demonstration, is often applied in language and philosophy (Malcolm, 1954; Wittgenstein, 1953); it is considered particularly effective in the clarification of semantics. In the context of healthcare ecosystems, FHIR can be regarded as a language to encapsulate local health data for cross-institutional exchange. Semantic ambiguities are generated when implementers have contrasting understandings of the FHIR’s lexical definition, leading to inconsistencies in its use. Therefore, this paper considers the clarification of FHIR’s meanings as understood by an implementer via the illustration of FHIR-represented data in local health information systems as examples of inconsistency between implementers.

In this research, the ostensive approach is a process of demonstrating the way in which FHIR is used by implementers to represent the healthcare services supported by local health information systems (HISs). Thus, this paper explains a healthcare service through the following steps:

1. explaining its lexical semantics by use of an FHIR knowledge graph;
2. explicating semantics by highlighting explicit correspondences between FHIR and local data attributes, and
3. showing examples of these attributes by describing the values of attributes that are stored in local datasets

The above three actions are carried out by the Semantic Engine, which is composed of a core and peripheral knowledge graphs. The core is the FHIR knowledge graph, and the peripherals are the nodes and their relationships, which reflect the correspondences between FHIR resources and the local database. The Semantic Engine has the capacity to:

1. provide semantic interpretation by displaying nodes and their relationships, and
2. retrieve relevant data from multiple local databases, map these data back to the nodes, and assemble the data according to the relationships between nodes.

The Semantic Engine can be regarded as a ‘switch’ within HISs to facilitate semantics interaction; it provides the denotation of healthcare concepts by a topology of nodes and corresponding examples to answer semantic queries from heterogeneous systems within healthcare ecosystems. Therefore, semantic interoperability can be enhanced through the sharing of consensus ontology and examples, while patient-centred care can be supported by the proposed ostensive healthcare information architecture.

This paper will illustrate two example cases of proposed ostensive information architecture functionalities. The first shows the way in which the Semantic Engine responds to semantic queries, while the second demonstrates how it retrieves data from heterogeneous databases to enable cross-institutional data sharing. Overall, this study provides a new perspective on the solution of semantic ambiguity in terms of information architecture. Further, the proposed ostensive information architecture naturally separates semantic explanation and data storage, which is an option to ensure data privacy and enhance data security.

This paper is structured as follows: Section 2 introduces the interoperability supported by FHIR and addresses its limitations in this respect. Section 3 reviews the available related work, while Section 4 explores the root cause of semantic ambiguity in FHIR implementation, comparing the lexical and ostensive approaches from a theoretical perspective. Section 5 describes the proposed ostensive information architecture, the functions of the Semantic Engine, and the value it offers, and Section 6 demonstrates the effectiveness of the proposed information system architecture by employing the MIMIC III dataset and diabetes datasets. Section 7 presents a summary of the Semantic Engine’s applications, discusses the study’s limitations, and provides some suggestions for future research directions, followed by the conclusions of this research.

## Interoperability Addressed by FHIR and its Limitations

FHIR is proposed by HL7 International to improve the interoperability of systems in the healthcare domain and to facilitate information exchange between the stakeholders of healthcare ecosystems. FHIR is an open suite of software specification and implementation, comprising two elements: information models entitled ‘*resources*’, and a specification for the exchange of these resources. The goal of FHIR is to render all health data accessible to large-scale analytics in order to improve the quality of healthcare services.

In contrast to the earlier standards of HL7 v2 and v3, FHIR is likely to rapidly gain attention from the sector because it actively embraces Internet technologies and offers advantages such as agility, fast iteration, and low learning costs (Bender and Sartipi, 2013, Zong et al., 2021, Xu et al., 2020, Leroux et al., 2017), with additional support for mobile applications (Mandel et al., 2016, Bender and Sartipi, 2013, Sayeed et al., 2020). FHIR adopts a RESTful API which facilitates interactions and represents data in the currently popular JSON (JavaScript Object Notation) format, in addition to the EDI and XML formats provided by the earlier standards. FHIR provides a set of standards with established patterns to improve interoperability among a wide range of systems and devices that transcend EHR (Electronic health record) systems. FHIR for heterogeneous healthcare information systems is akin to the TCP/IP standard for the Internet. FHIR significantly reduces the difficulty of the transformation of incumbent information systems and its implementation compared to OpenEHR and the previous versions of HL7 (Bender and Sartipi, 2013).

In 2018, six Internet giants, namely Amazon, Google, IBM, Microsoft, Oracle, and Salesforce, jointly committed themselves to the elimination of interoperability barriers in healthcare by adopting FHIR as an exchange standard (Information Technology Industry Council, 2018). FHIR is selected as the basis of this research because it has been adopted as the national standard across all hospitals in the United Kingdom (UK) (NHS, 2020) and has also been widely adopted in other sectors.

In addition to the RESTful interface, FHIR *resources* can be exchanged through the paradigms of document, messaging and services; these comprise the three types of *resource* collections serving different purposes (McKenzie, 2016). For current solutions, FHIR is usually adopted as a front-end server, expressing the local healthcare data with the term ‘*resources’*; it provides an HTTP/REST interface for applications by developers to access data (Saripalle et al., 2019). Heterogeneous databases mutually communicate through their front-end servers. These FHIR servers are oriented towards each other, establishing an unimpeded network of intercommunication through RESTful APIs at the technical and syntactical level. The semantic interoperability between heterogeneous databases is theoretically ensured by FHIR *resources*, which constitute unified information models to ensure that all agents communicate via the same discourse system.

The fundamental units of FHIR that represent clinical information are *resources*, which are information models featuring a set of pre-defined properties for a specific aspect of the domain. For example, the *resource* representing an individual patient has attributes including name, gender, address, and date of birth. Effectively, a *resource* can be identified as a schema, which describes all of the relevant attributes of a conceptual entity. Currently, FHIR R4 defines 146 types of *resources* within five categories; these are *Foundation (30), Base (26), Clinical (39), Financial (16), and Specialised (35).* FHIR consolidates all categories of data with these pre-defined *resources,* which are already in use or will be used in HISs. FHIR, as a sign system, offers a defined lexical space in which clinical concepts, healthcare services, and FHIR resources are utilised.

The widespread adoption of FHIR has led to an increased debate on the limitations of semantic interoperability; Kubick (2016) and Kraus (2018) discuss the semantic ambiguity introduced by the implementors due to different combinations of FHIR *resources* being used to explain the same healthcare service. When FHIR is adopted as an ‘interpretation wrapper’ in a healthcare ecosystem for information exchange, all parties to it are able to choose FHIR *resources* to represent their healthcare services. In consequence, different institutions may not be able to interoperate due to the inconsistencies in the use of FHIR *resources*; semantic ambiguity in communication based on FHIR *resources* is introduced and amplified by the interactive process.

Three types of semantic ambiguity have been identified in FHIR implementation, with the first caused by the insufficient rigour of the FHIR specification. Beale (2019) contends that the inconsistency in the definition of FHIR produces semantic ambiguity. The following examples have been found in FHIR v4.3.0:

- **Same semantics with different lexical names**

*Dosage* in *Medication Statement* has the same meaning as *Dosage Instruction* in *Medication Dispense*. The three elements, *Location.hoursOfOperation, Healthcare Service*.*Available Time*, and *Slot.start*, are different names, although they appear to designate the same thing.

- **Same lexical name with different semantics**

The ‘substitution’ in *Medication Request* and *Medication Dispense* describes two different actions.

- **Same lexical name and semantics, but different data structure**

The ‘status reason’ in *Medication Request* is defined as a Single-valued attribute; in *Medication Administration,* it is a container attribute, and in Medication Dispense, it includes two sub-elements.

These imprecise definitions inevitably lead to misuse or inconsistency in the implementation of FHIR; further, the FHIR specification involves terminology in a variety of fields and is relatively complicated. The lexical definitions do not have the capacity to guide implementers to precisely match FHIR resources to idiosyncratic local databases because it is impossible for the FHIR specification to describe all mapping scenarios; this is an inherent flaw of the lexical approach, which is discussed in Section 4 from a theoretical perspective.

The second type of semantic ambiguity is introduced by FHIR *extensions*. As the 80/20 rule of FHIR *resources* (HL7 International, 2019) is adopted to avoid overlap and redundant definitions of resources, lesser-used terms can be freely defined by implementors in the format of *extension of resources*, which accounts for 20% of clinical terminologies. Because FHIR unifies healthcare resources but lacks explicit contextual constraints, the 80/20 rule enables an institution to define its own *extensions* for the same healthcare service. An issue of this nature both causes barriers to information exchange and also obstructs medical discoveries based on cross-institutional data analysis (Dolin et al., 2018). Semantic interoperability particularly deteriorates when *extensions* of *resources* are used to deal with specialist health data.

**Fig. 1:**
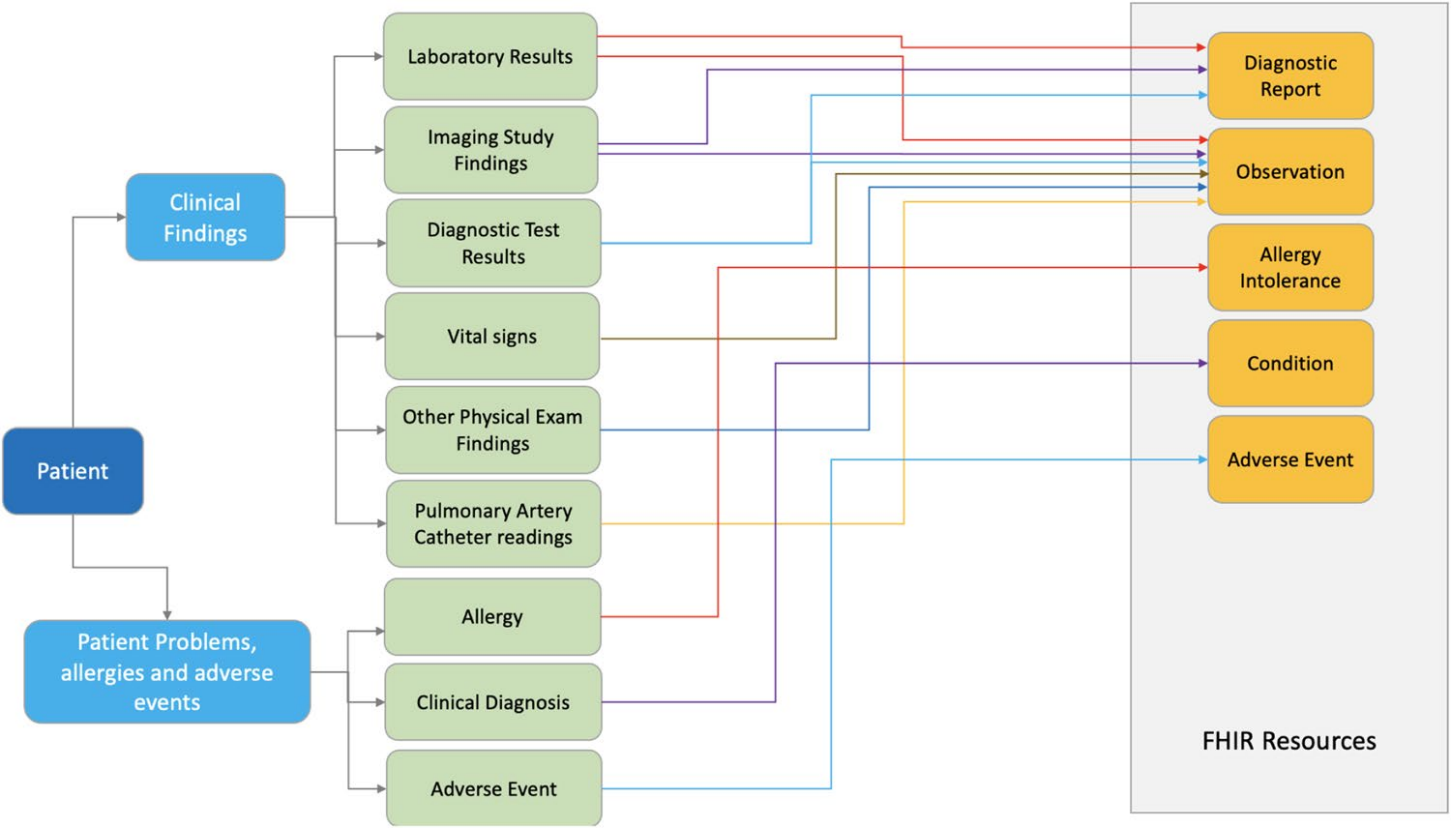
An example of correspondence between clinical actions and FHIR resources (HL7, 2022)

The third type of semantic ambiguity is due to the freedom and flexibility FHIR offers implementers; they can employ FHIR *resources,* or combinations of them, in order to interpret healthcare services, even though some may not be mature and/or stable, which leads to semantic ambiguity. FHIR v4.3.0 defines 139 *resources*, of which 100 belong to non-clinical categories. The number, which increases with every release, grants a substantial degree of freedom to implementers to use these *resources*. For example, in Fig. 1, the *resources* of ‘*observation*’ can be combined with other *resources* to represent laboratory results, imaging study findings, diagnostic test results, vital signs, and other physical examination findings. These are the combinations of FHIR *resources* suggested by HL7 (https://www.hl7.org/fhir/resourceguide.html). The potential misuse of *resources* occurs when healthcare data are beyond the scope of HL7-suggested combinations. Additionally, FHIR implementers can tailor FHIR integrations to specific business needs, resulting in multiple customised resource collections occurring between different systems (Dolin et al., 2018; Jiang et al., 2016). The interoperability issue caused by diversified FHIR collections is recognised by HL7 International (2022).

This research aims to demonstrate these semantic ambiguities generated in FHIR implementation and seeks to decrease their occurrence by exposing the correspondences between FHIR resources and the attributes of local datasets. When the custom use of FHIR is demonstrated to other parties within the healthcare ecosystem, stakeholders can select its optimum interpretation and promote it to the standard of the whole ecosystem.

## Related Work to Improve FHIR Compliance

Regarding the three types of semantic ambiguity discussed in Section 2, HL7 launched an office website (http://hl7.org/fhir/registry/) to manage *extension* publications. Once an *extension* defined by implementers is approved by HL7, it is shared with all FHIR users through an official channel; this centralised management method effectively unifies and standardises custom extensions. However, the use of *extensions* faces the problem of interoperability caused by implementers' contrasting understandings of lexical definitions. McClure et al. (2020) propose a framework to guide harmonisation among multiple FHIR users in terms of terminology, data elements, measure clauses, and measure concepts. Tute et al. (2021) take a similar approach, proposing a data quality assessment method for the support of collaborative governance.

The approach of ensuring FHIR conformity through review processes is usually costly in terms of time and labour. Sayeed et al. (2020) take an alternative approach, proposing an application that automatically merges patient-generated health data, represented by FHIR resources, into EHR. This approach is effort-effective but its scope is limited to patient-generated health data, and it does not cover electronic health records, which are the most complex aspect of healthcare ecosystems. Pfaff et al. (2019) contribute mapping scripts for the interpretation of medical data with FHIR *resources*; in their study, the data from the Integrating Biology & the Bedside (i2b2), the Patient-Centred Outcomes Research Network (PCORnet), and the Observational Medical Outcomes Partnership are automatically encapsulated by the FHIR. However, the script compatibility issues caused by the idiosyncrasies of local data sources persist. In their framework, the adaptation of a local database to the mapping script is allocated to the local database layer; therefore, the inconsistency of using FHIR *resources* caused by different implementers remains unresolved.

Another approach is to leverage the national effort to harmonise the FHIR resources for medical data representation across hospitals. The Medical Informatics Initiative (MII) and local data integration centres (DICs) in Germany collaborate to standardise COVID-19 data in FHIR profiles through another set of models, i.e., the German Corona Consensus Dataset (GECCO). Using FHIR, GECCO defines 83 data elements and has been extended to all hospitals nationwide (Rosenau et al., 2022). The United Kingdom adopts the same approach and proposes FHIR UK Core (NHS, 2020) to enable consistent information flows across borders. However, the disadvantage of this approach is the lack of agility and the high cost of upgrading.

In industry, a more straightforward approach is adopted; a developer collaboration and publishing platform (Firely, 2015) plays the role of coordinator and facilitator among developers to improve the conformance of FHIR, constituting a loosely-regulated approach. The above approaches have their own advantages and disadvantages; this study seeks to develop a low-cost, and high-efficiency method by which to ensure FHIR conformity.

Table 1 summarises the benefits and constraints of existing FHIR compliance solutions in terms of cost, efficiency, implementation difficulty, and application breadth. The suggested ostensive information architecture has clear advantages over current solutions.

**Table 1:** Comparison between FHIR Compliance Solutions

Solution	Time and cost	Efficiency to decrease ambiguity	Easy to Implement	Scope of application
HL7 official website (http://hl7.org/fhir/registry/)	Low	***Low***	Easy	Wide
A framework for harmonisation (McClure et al., 2020; Tute et al., 2021)	***High***	High	***Hard***	Wide
Automatic tools (Pfaff et al., 2019; Sayeed et al., 2020)	Low	High	Easy	***Narrow***
FHIR resources harmonisation national wide (NHS, 2020, Rosenau et al., 2022)	***High***	High	Easy	Wide
A developer collaboration and publishing platform (Firely, 2015)	Low	***Low***	Easy	Wide
An ostensive information architecture	Low	High	Easy	Wide

In view of the necessity for FHIR to use a consensus-based approach, this study considers the related work of ontology used as an artefact to promote the harmonisation of health information systems. Ontology artefacts play a critical role in the fields of medical terminology unification, cross-medical protocol interoperability, and information exchange between heterogeneous systems for healthcare services. In summary, relevant ontologies comprise the following three types:

- **Ontology for terminology: representing terminological and taxonomic aspects of medical knowledge**

Ontology has been used to unify the medical interpretations in order to establish an agreement on medical terminologies in diverse clinical systems, such as LOINC and SNOMED-CT. From this perspective, terminology ontologies are the pre-defined agreements designed to standardise the language of a domain, providing each term with a precise meaning and a specific granularity. Therefore, terminology ontologies lay the foundation for the exchange of medical information.

- **Ontology as a bridge between two systems: representing the relationships between terminology ontologies**

Regarding the clinical concepts that comprise the medical terminologies defined by different standards, ‘bridge’ ontologies are adopted to facilitate information exchanges between terminology ontologies and are employed to unify the definitions of clinical concepts. Ryan (2006) interconnects HL7 v3 and SNOMED-CT through ontology matching, and Bodenreider (2008) uses the same method to enable SNOMED-CT to understand the laboratory test result coded in LOINC. Those similar operations facilitate semantic interoperability between heterogeneous coding systems and also support the integration of dispersed health information systems (Plastiras et al., 2014).

- **Domain ontology: providing a common knowledge base for healthcare ecosystems**

Compared to the healthcare domain ontology proposed by individual researchers or national institutions, the FHIR-based ontology has evident international influence and the advantage of wide promotion. To encourage FHIR’s adoption, the following studies propose methods for the transformation of healthcare data into the corresponding HL7 FHIR structure (Jiang et al., 2017; Kiourtis et al., 2019). A significant amount of research effort has been devoted to the improvement of FHIR coverage scenarios. Beredimas et al. (2015) propose an OWL (Web Ontology Language) ontology that defines the primitive and complex data types of the FHIR framework and the validation rules to enable FHIR to express data information externally to traditional medical databases.

El-Sappagh et al. (2019) extend FHIR to the telehealth scenario, introducing real-time sensor data into the historical EHR medical data with the aim of providing more comprehensive patient data to clinical decision support systems. Similar works have been carried out by Peng and Goswami (2019), combining data generated from the Internet of Things (IoT)-empowered smart home devices to EHR; meanwhile, Mavrogiorgou et al. (2019) collect multi-dimensional data reflecting patients’ health. This type of research (Moreira et al., 2018; Wagholikar et al., 2017) extends the application of FHIR to a broader range of medical data, promoting the wider adoption of FHIR.

Literature review provides evidence that ontology artefacts are widely-adopted, with the aim of improving data harmonisation and accessibility, and FHIR-based ontology is a mainstream approach to contend with the ever-increasing complexity of healthcare ecosystems. Thereby, this research study explores the FHIR conformity solution on the basis of the FHIR ontology artefact. The next section delves into the root cause of semantic ambiguity in FHIR implementation.

## Information Interaction through Lexical and Ostensive Approach

By investigating the practice of information management and human communication, we recognise that the fundamental cause of semantic ambiguity generated in FHIR implementation lies in using signs to represent objects. A sign can be anything that is interpreted as a substitute for something else (Eco, 1979), particularly in human communication. In semiotics, researchers examine information interaction through the study of signs and their effect on the human actors involved. Multiple semiotic theories hold different stances in epistemology and have laid different cornerstones in communication. They profoundly impact the fields of informatics (Liu & Li, 2015; Liu et al., 2010), information systems (Baxter et al., 2018; Brödner, 2019), knowledge management (Holzinger et al., 2014) and artificial intelligence (Chartier et al., 2019; Staab, 2019; Targon, 2018).

**Fig. 2:**
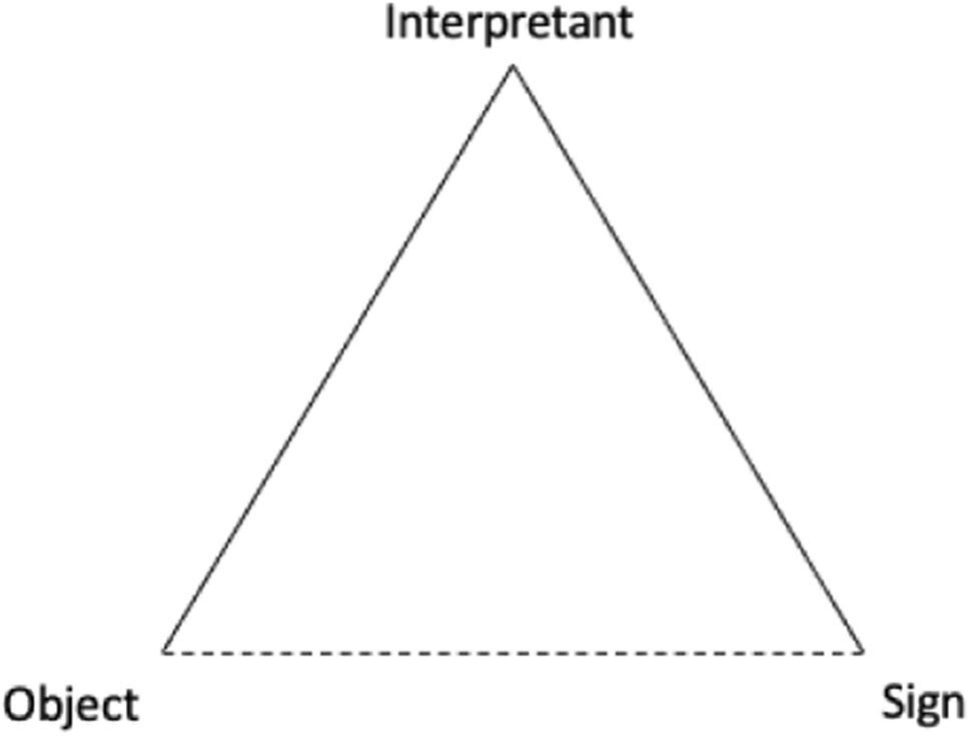
Peirce’s triadic model (Peirce, 1958)

Saussure’s theory of signs originated with the thought of a dichotomy. He believed that a sign links a signifier and a signified, which may exist in material form or as a concept. The signifier in his theory is something that explicitly exists and can be distinguished by human senses (Leeds-Hurwitz, 1993). Peirce reckoned that the existence of an interpretant is critical and must be introduced in the process of making sense of a sign, which he terms a semiosis. An interpretant directly connects a sign and an object, while the sign and the object are linked by a dotted line (Fig. 2) in Peirce’s triadic model. The dotted line in the figure indicates that the correspondence between the sign and the object is not objectively determined but is dependent on the context and purpose of the communication and hence subject to personal interpretation. The interpretant can be regarded as the effect of such a sense-making process (Chandler, 2017) through the use of signs in different contexts or for different purposes (Liszka, 1990; Savan, 1987). Therefore, between a sign and an object, there is no strict one-to-one correspondence as suggested in Saussure’s model; although most specifications for information sharing adopt the Saussurean model of static mapping between lexicons and objects, including FHIR.

Peircean semiotics emphasises the effect of using signs in context (Staab, 2019). By emphasising the subjectivity in the mapping between the sign and the business context in which the sign is used, the triadic model of semiosis offers a theoretical basis for an ostensive approach to pinpoint the meaning of the sign (i.e., semantics) and its effect on the sense-making of the sign (i.e., pragmatics).

To deal with the possibility of one-sign-multiple-objects, the ostensive approach is introduced to explicate the sign-object correspondence via direct demonstrating actions and examples. In such a way, semantic ambiguity is resolved, especially when complex signs such as FHIR are involved.

**Fig. 3:**
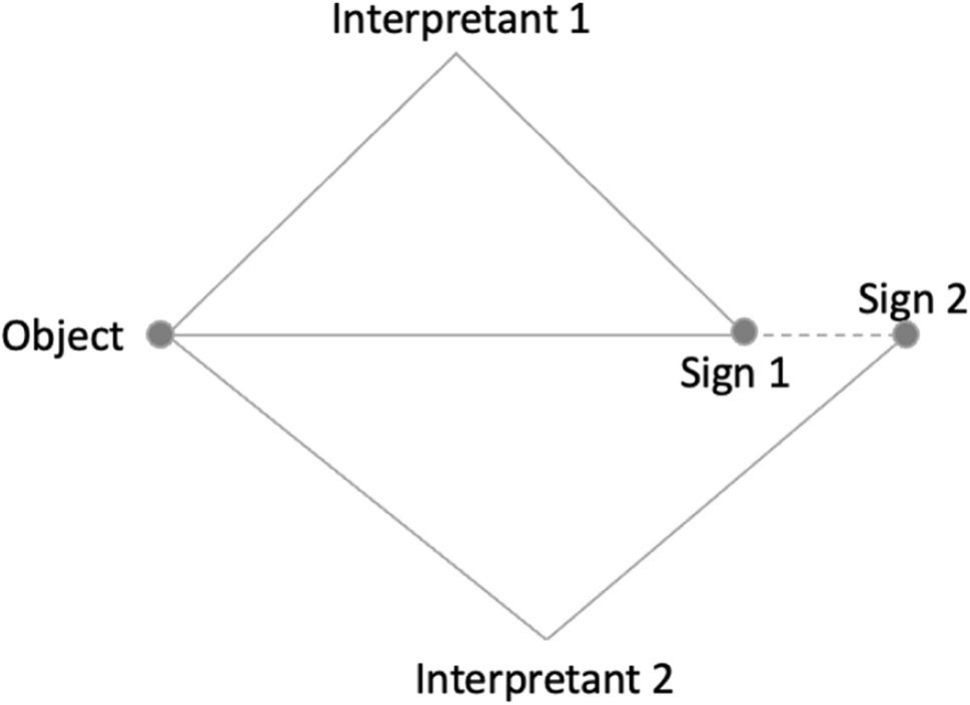
Peirce’s triadic model

The FHIR specification uses the lexical approach to explain the definitions of *resources*. In other words, FHIR interprets the meanings of *resources* in language, which can be understood as the ‘sign’ (as illustrated in Fig. 2). In the context of FHIR implementation, different implementers may have contrasting understandings of the FHIR definition, leading to the same FHIR resource being used to explain different clinical data. Semantic ambiguity is created when many interpreters illustrate the same concept with contradicting signs. The ambiguity of "same semantic with different lexical names" is depicted in Fig. 3. Similarly, the ambiguity of ‘same lexical name with different semantics’ occurs when the same sign is mapped to multiple objects by different interpretants. Just as Dolin et al. (2018) addressed, the primary challenge of FHIR adoption is to transform multiple distributed local datasets into consistent FHIR formats.

Therefore, this paper proposes an ostensive approach as a complement to the lexical approach in order to reduce the semantic ambiguity introduced by the contrasting understanding of the FHIR definition.

## An Ostensive Approach of Elucidating Semantics

This research study adopts FHIR as the grounds on which to explain the concepts in the healthcare domain. In response to the limitations of FHIR, an ostensive approach is proposed that provides clinical data as examples to further explain the semantics defined by FHIR, along with the understandings of different implementors.

Thus, a knowledge graph is constructed on the basis of FHIR, and the FHIR knowledge graph is extended in order to connect attributes stored in local databases; this is termed ‘FHIR-centric knowledge graph of the Semantic Engine’, enabling semantic elaboration and reasoning, and the clinical data stored in heterogeneous local information systems can be retrieved by the Semantic Engine. In summary, the core of the Semantic Engine is the FHIR knowledge graph; the peripheral consists of attribute nodes in the local datasets. The correspondents between the FHIR resource nodes and local attribute nodes are connected by ‘mapping’ lines. Due to the fact that attribute nodes are defined by local implementors, it is difficult to name nodes consistently, which may lead to ambiguity.

The query statements sent by clients to the Semantic Engine reflect their understanding of the FHIR specification through the lexical approach. The data in response to the request ostensibly exhibits the data providers’ understanding of FHIR specifications. If there is mismatching between the clients and data providers in terms of the understandings of FHIR, the data in the response can help the user to comprehend the gap. In summary, the proposed information architecture helps users to comprehend the semantic ambiguity produced by the lexical approach through the ostensive examples.

In general, the Semantic Engine is responsible for the processing of all semantics-related tasks. For example, the meaning of a node can be elaborated by the nodes connected with it and their relationships; effectively, the topology graph of this node discloses the node’s meaning. Semantic reasoning can be conducted through analysis of the relationships between nodes, for example, the shortest path between two of them.

**Fig. 4:**
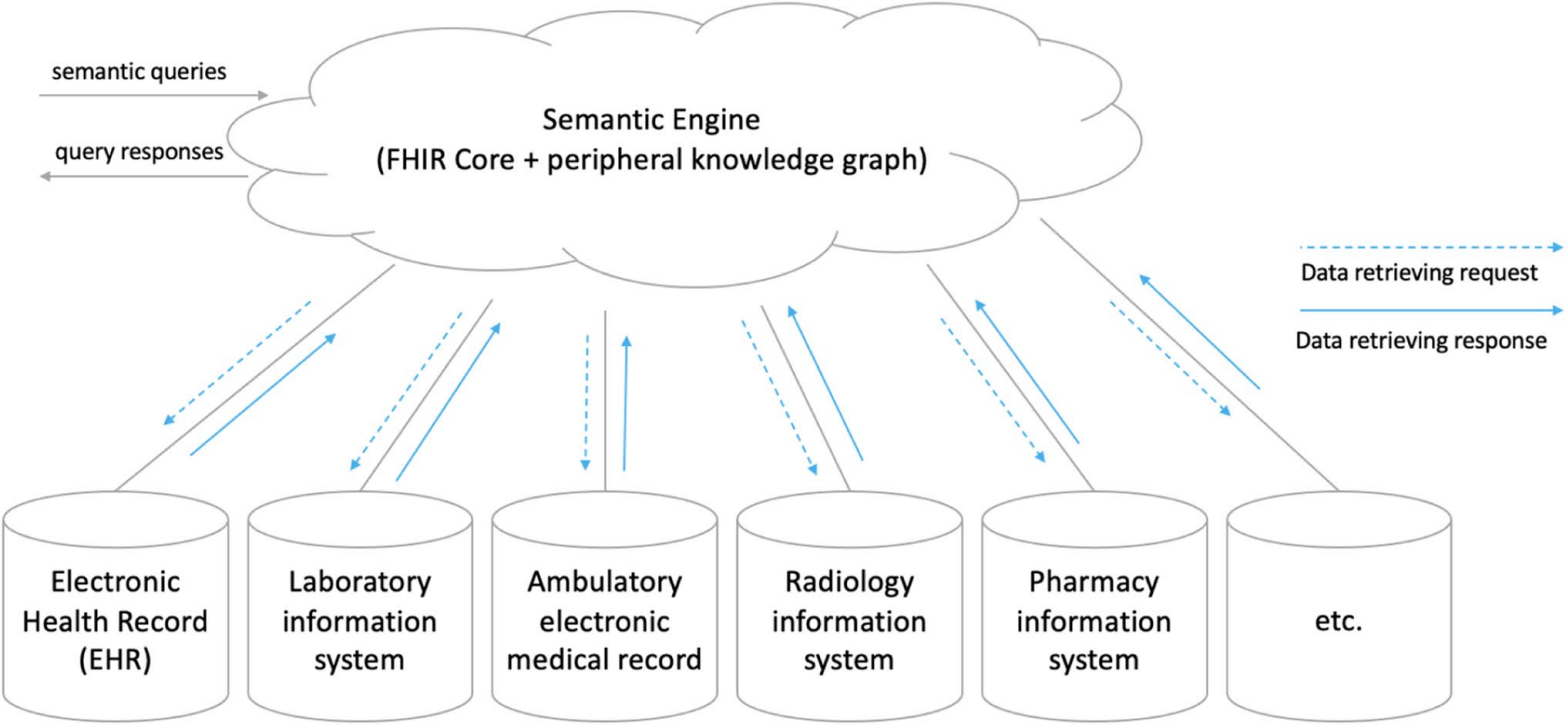
A semantics-data separated information architecture

This paper proposes this semantics-data separated architecture for HISs to support semantic interoperability (Fig. 4), which can be regarded as a federated architecture. The Semantic Engine maps and integrates data from autonomous component database systems. The federated architecture (Wallender et al., 1979) is a common approach to integrate data from dispersed databases (Batini et al., 1986) and is adopted in the healthcare domain (Dusetzina et al., 2014).

The key purpose of this design is to employ FHIR in a computational method that leverages the advantages of the knowledge graph to process semantics, and takes privacy and security concerns into account. The separation of semantic processing and data storage can reduce the problem of data privacy leakage caused by the unified storage of data, and access mechanisms based on authorization further guarantee data privacy; this is discussed in Section 7.

In summary, the Semantic Engine has two main functions:

1. the enhancement of semantic interoperability across dispersed health information systems by feeding back the JSON file to show the semantic definitions in FHIR along with the understandings by implementors; and
2. the elimination of semantic ambiguity by providing corresponding examples in the form of data stored in different local health information systems.

### An Ostensive Information Architecture

**Fig. 5:**
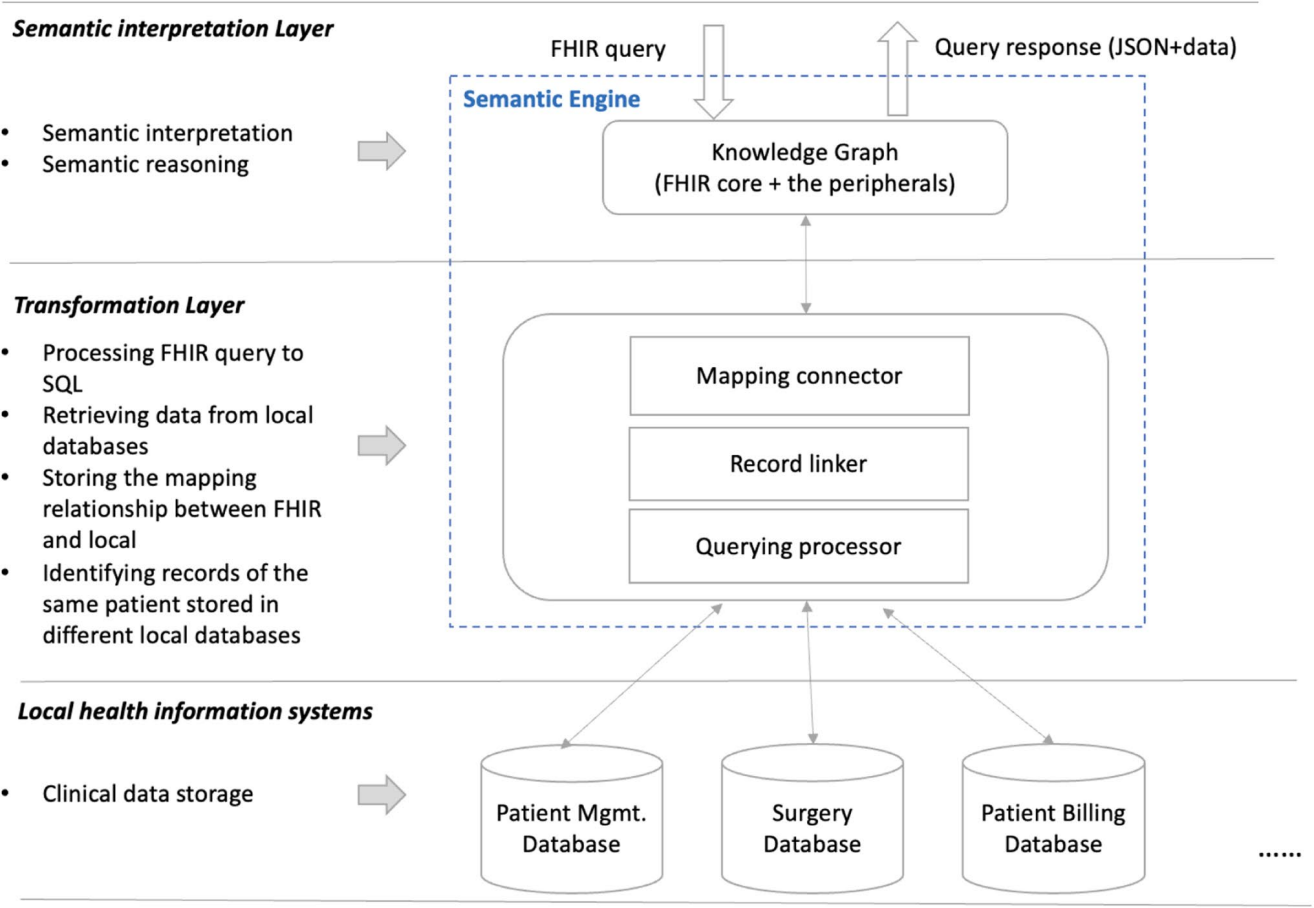
An ostensive architecture of HISs

On the principle of separating semantic processing and data storage, this study positions FHIR in health information systems. In contrast to the use of FHIR as a standard protocol for the transformation of local databases for information exchange, FHIR is abstracted from the front-end of each local information system and unified at the logical top level of the entire health information system (Fig. 5).

This study’s proposal simplifies the architecture of health information systems by centralising the semantic interpretation layer in order to avoid the ambiguity caused by different interpretants of FHIR, which means that the correspondence between the data provided by the specified primary database and FHIR *resources* is a system-wide standard, and other systems that differ from the standard definitions should follow the semantic interpretation of the primary system. For example, patients’ names and addresses could come from multiple clinical systems, but the patient registration system is usually taken as the primary system. When a query requires the ICU (intensive care units) information of a patient to be provided by the Semantic Engine, this will feed back the name and address from the patient registration system and the relevant ICU information from the intensive care information system, with the name and address of this patient stored in the intensive care information system in different formats being ignored. The centralised semantic interpretation layer can be deployed on the cloud to solve the problem of access bottlenecks caused by multiple requests.

Through setting the master–slave relationships between dispersed systems, the system-wide semantics are now unified; in other words, regarding a piece of data to describe a certain patient attribute, there is only one mapping relationship between FHIR and local clinical information systems within an entire ecosystem, even though there are multiple databases storing the same patient attributes. When two peer hospitals make inconsistent use of FHIR *resources*, the two different mapping methods are represented as two external graphs to the FHIR knowledge graph. The local implementers of both hospitals can establish a consensus by comparing and selecting.

The architecture of the proposed HISs is shown in Fig. 5. To support data retrieval, the Semantic Engine comprises two main elements: FHIR knowledge graph and transformation components.

This architecture contains three layers in order to respond to the FHIR queries; the semantic interpretation layer is a FHIR knowledge graph, which provides an explanation of semantics in lexical definition by nodes and their relationships. The transformation layer works with local health information systems to provide the semantics by example, constituting the data stored in heterogeneous local systems. The **mapping connector** in the transformation layer matches data with FHIR resources, generating conflict alerts if and when data inconsistencies are detected. For example, an alert occurs if a date of birth has been assigned to two data sources through schema matching (Section 5.2.2) or the same concept has been interpreted by different FHIR *resources*. Therefore, the mapping connector consists of several sub-components.

An explanation is provided in Section 6.2.2. of the functions that convert the data from two data sources into a unified FHIR-defined format in the **Record linker,** which combines the records of the same patient from different databases. For example, the record linker can recognise the records for a patient in a hospital’s billing system and the claim management system of an insurance company, associating the two records. The **querying processor** translates queries from the Semantic Engine and obtains data from local databases. The bottom layer represents the local healthcare information systems where the clinical data are stored.

**Fig. 6:**
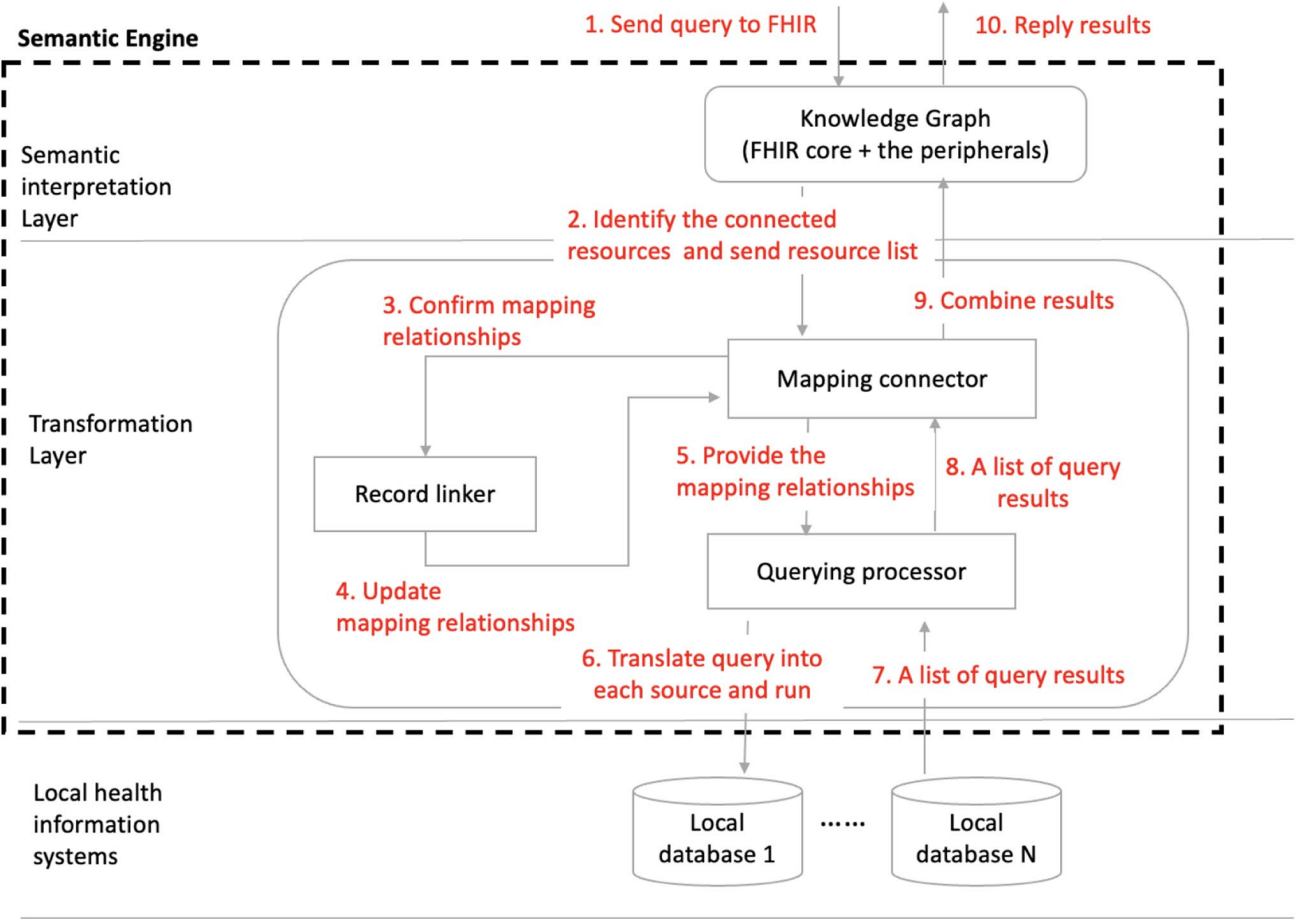
Semantic query processing flow

Figure 6 illustrates the processes of a semantic engine dealing with a semantic query. The FHIR knowledge graph plays a critical role as a user interface and semantic interpreter. Ten internal steps (shown in Fig. 6) include redirecting user queries to different local databases, generating query statements, collecting query results, and merging them to return responses to users. Nie and Roantree (2019) address the question of how to merge the records of different aspects of the same object when they are stored in multiple databases. In this study, the patient profile can be taken as a key variable by which to conduct the record linkage.

### Semantic Engine

As previously mentioned, the core of the Semantic Engine is the FHIR knowledge graph; this study uses Neo4j (Lal, 2015) for its development. In order to facilitate data exchange between dispersed information systems, the local data require connections to the Semantic Engine. This research transforms the properties of local data into these property nodes; values of local data are retrieved as examples to further render the semantics explicit. This Semantic Engine can support semantic interpretation, semantic computing, and semantic reasoning. This research study focuses on the function of semantic interpretation, which explains concepts to the queries. The details of how the Semantic Engine is structured based on FHIR schema are shown below, along with how local data connections to the Semantic Engine are implemented.

####  The Construction of an FHIR Knowledge Graph

**Fig. 7:**
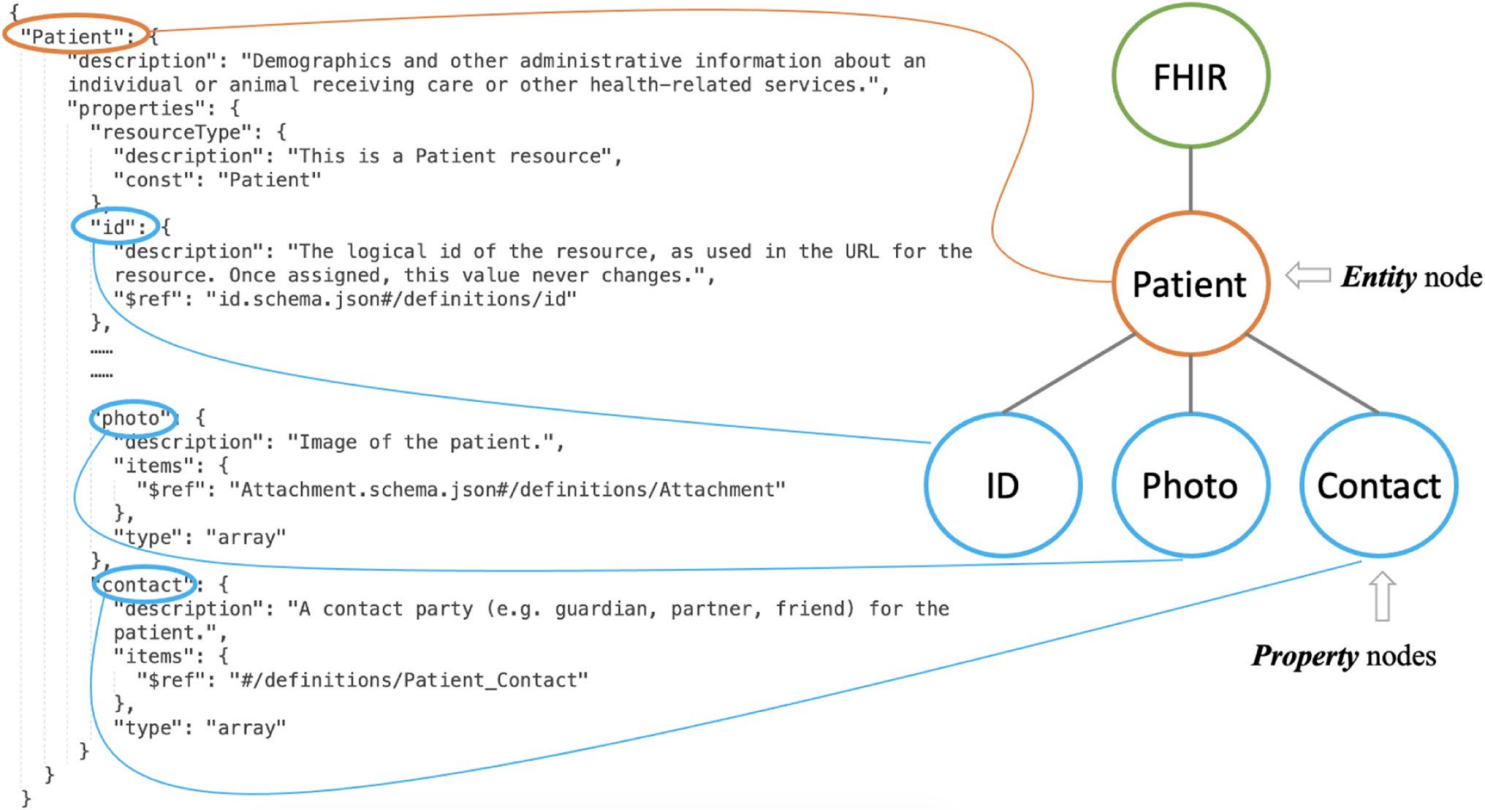
FHIR – Graph mapping

The JSON representation of an FHIR schema is used to construct the knowledge graph, with each defined entity becoming an ***Entity*** node. Each property of the defined entities occupies a ***Property*** node. Relationships between entities that are defined within the JSON schema become edges within the knowledge graph. Figure 7 details the construction of a knowledge graph from the FHIR JSON schema.

This JSON is parsed and converted into a series of mappings which are imported into a Neo4j graph database with the top-level object *“Patient”* becoming an *Entity* node and all sub-objects become *property nodes* within the graph. Once the data is inside Neo4j the query language Cypher (Lal, 2015) can be used to view all information relating to a Patient. The software to generate the knowledge graph is available here (Guo et al., 2023).
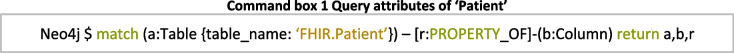


Similar Cypher commands can be used to query other *resources*. Since the *resources* are interconnected, the FHIR knowledge graph can be constructed.

#### Schema Matching

This step is designed to clarify the correspondence between FHIR resources and local data. The knowledge graph of the Semantic Engine comprises a set of nodes, $\mathrm{N},$ and a series of edges, $E$. This knowledge graph contains not only the low-level mappings for individual data sources but further abstractions of these data, providing the capacity to semantically reason. For the remainder of this section, this paper focuses on the schema-matching and schema-mapping components, which are used to provide a basis for interoperability between healthcare systems. To map data stored in dispersed systems correctly to the Semantic Engine, each individual source must be understood in detail; this requires a graph model that can capture the complexity of this individual source.

**Fig. 8:**
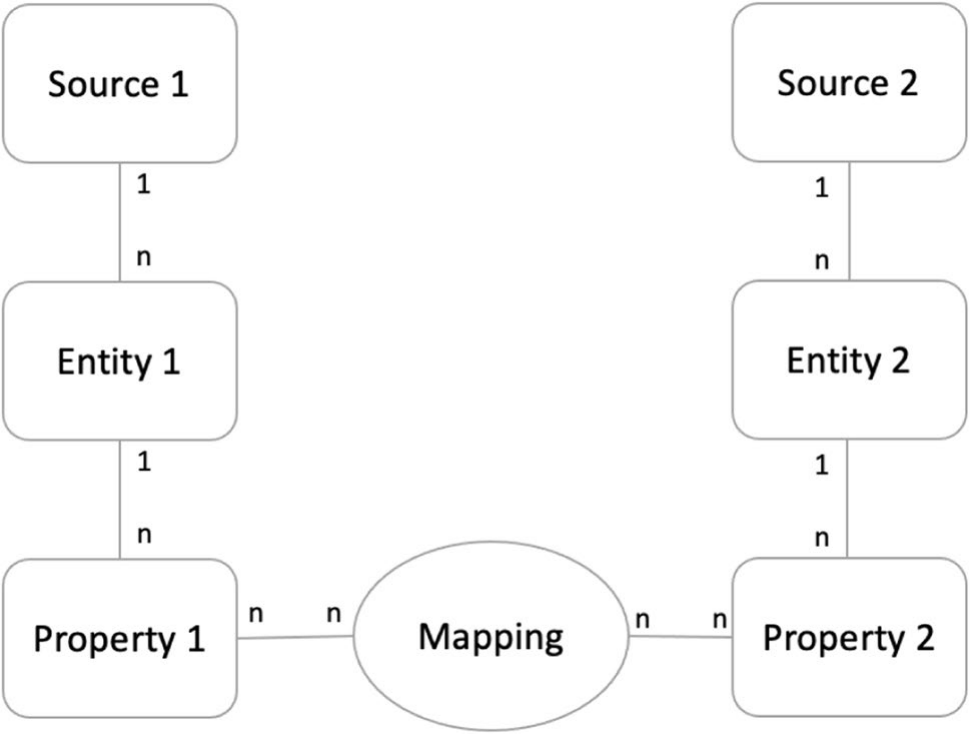
Graph Structure

Figure 8 illustrates, at a high level, the nodes and edges required to effectively provide a means by which to perform schema mapping. Nodes in the graph represent sources, properties, and mappings; edges are used to denote relations between them. Within the graph, there are four node types, specifically:

- ***1. Source*** denotes a particular data source, identifying the system from which the data are obtained. This node contains the connecting information for an individual source to facilitate communication with a particular mapping connector. 2. ***Entity*** relates to a particular entity from a data source; within a DBMS (database management system), these may correspond to tables. 3. ***Property*** refers to an entity’s attribute, such as the name of a patient, which corresponds to columns within an RDBMS (relational database management system); there is a ‘one-to-many’ relationship between an entity and its property. Finally, 4. ***Mapping*** denotes the way in which two properties between local data sources and the semantic engine may be related.

#### Mapping Data to FHIR

This study uses MIMIC III (https://mimic.physionet.org/about/mimic/) and a diabetes dataset (https://archive.ics.uci.edu/ml/datasets/Diabetes) as two local health information databases. MIMIC III consists of 26 tables with 58,598 instances covering 12 years intensive care data from 2001 to 2012. The diabetes dataset includes more than 250,000 records. In the process of mapping the data with corresponding FHIR *resources*, it is often the case that a concept defined by FHIR requires data from multiple MIMIC data tables for ostensive interpretation. Because MIMIC datasets are focused on intensive care medicine, many concepts defined by FHIR cannot be fully explained by MIMIC data, although it can be the case that two data records need to be amalgamated to match an attribute of an FHIR *resource* or a data record needs to be split into two segments to match the attributes of the FHIR resource. There is also a conflict between the index relationship between the MIMIC database and the FHIR resource, which occurs when querying the health information of an individual patient. Restrictions such as data types should follow the definition of FHIR and be guaranteed by the implementer.

When all datasets within a health ecosystem are matched with FHIR *resources*, it can be said that the health information relating to patients has been semantically connected. By this stage, any stakeholder in the health ecosystem can theoretically access all health information relating to a specific patient; therefore, patient-centred diagnosis, evidence-based medical research, medical insurance services, public health policy development, and such other healthcare-related services can be supported by this system.

In order to map data to FHIR, the *structural mapping information* of the data source, a set of *contextual mappings,* and a series of *transformation functions* are all required.

*Structural information* links entities and their properties within the graph, with each entity and property representing a node. The structural information is either derived from a supplied schema such as an RDBMS or, for flat files, manually supplied by a user. Once the structural information is converted into the graphical format, it can be mapped to the FHIR knowledge graph using the ‘Cypher’ command for processing. This is required in order to overcome differences in terminology and structural differences where one entity in FHIR may be composed of two or more entities within the local data source. This challenge resulted from the semantic ambiguity described in Section 2 and is the reason why this study sought to expose the inconsistent use of FHIR among its implementers.

The *contextual mappings* denote the context in which a specific data source is to be used; for example, the FHIR schema contains the concept of an “*observation*”, referring to medical observations, such as body weight or bone density. While this entity has wide usage due to its generic nature, specific data sources may focus only on a specific measurement. For the diabetes dataset, while it is an *observation* within the FHIR schema, it should only be queried if the user is requesting blood glucose levels.

This requires a mapping that can determine context; it can be achieved by embedding the semantics of the mapping within a *mapping* node. When mapping across data sources, the data may require semantic augmentation in order to ensure accuracy. An example is data, which provide values for the same entity, such as blood glucose levels, but are represented by differing units of measurement. These inconsistencies are overcome by using transformative functions embedded within the mapping nodes linking two properties.

In this research, the MIMIC data are converted into a graphical format using the relational schema and then supplemented with manual mappings to FHIR supplied in CSV format for batch processing with Cypher. The diabetes data are a series of flat-files; this representation therefore does not contain the necessary structural information, which was provided by a domain expert. In addition, the diabetes dataset has low dimensionality, requiring the provision of additional *contextual* mappings in order to accurately map the data to FHIR. The schema and data mapping are performed manually in this research, whereas in industry, developers can use tools to convert local data into the FHIR format in batches (Kiourtis et al., 2019). Regardless of the method used by the implementer, the purpose of this step is to illustrate the corresponding relationships between FHIR and local data in node-edge format.

## Enhancing Semantic Interoperability with the Semantic Engine

In this section, two case studies are conducted in order to explain that the proposed ostensive information architecture can:

1. decrease semantic ambiguity by showing the data values and their context, and
2. synthesise data from disparate systems with the aim of achieving patient-centred diagnosis.

### Ostensive Approach to the Enhancement of Semantic Interpretation

FHIR v4 defines 146 types of resources to describe the concepts within the healthcare domain; all resources are represented in JSON format, and naturally have sufficient feasibility to be represented by a knowledge graph. Because Neo4j enables semantic searching and reasoning, the meaning of a concept such as ‘Patient’ can be easily understood through the property node and its relationships. For this reason, the FHIR knowledge graph is termed a ‘core Semantic Engine’. The lexical definition can be searched on the Semantic Engine, while the ostensive examples can also be retrieved by it. The following example illustrates the way in which the ostensive approach supports the reduction of semantic ambiguity.

**Fig. 9:**
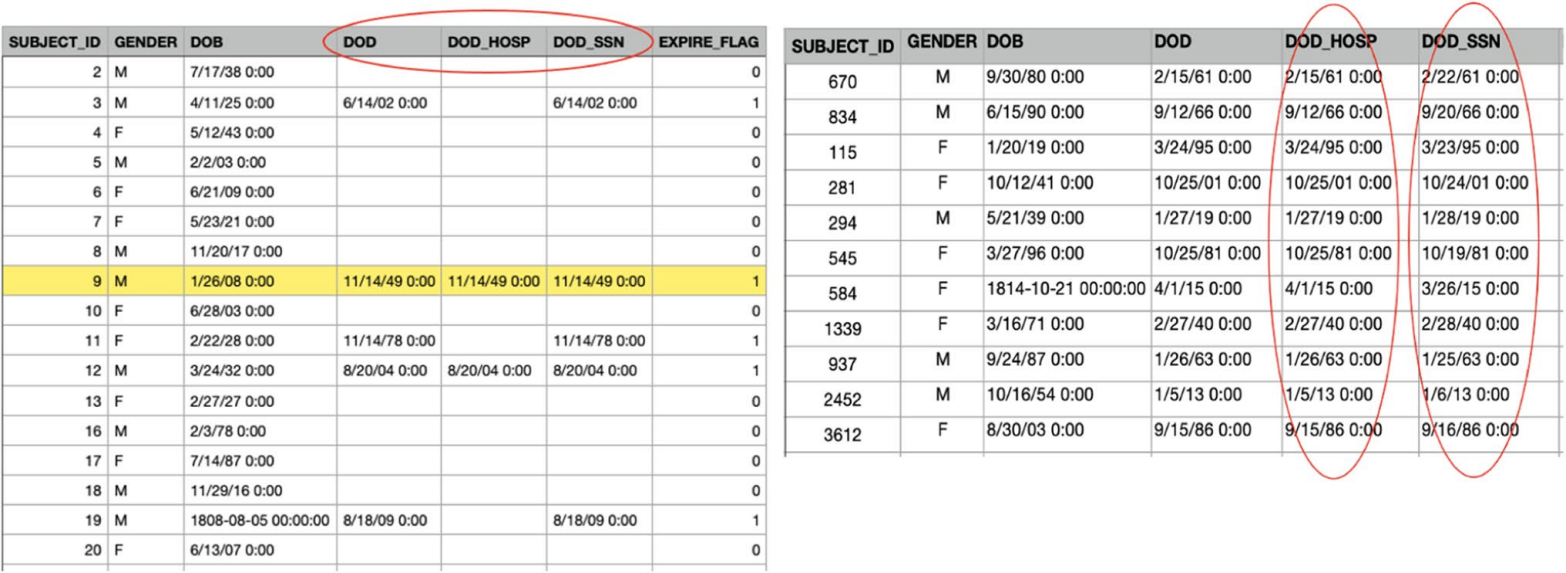
Patient table in MIMIC data sets

In FHIR, for example, one of the properties of ‘patient’ is ‘DeceaseDateTime’. Because FHIR has not clearly defined the concept of date of death with context, the possibility of the introduction of semantic ambiguity occurs. In MIMIC datasets, two tables reflect the content of ‘death time’. There are three relevant columns in the patient table (Fig. 9): DOD, DOD_HOSP and DOD_SSN.

DOD_HOSP indicates the date of death stored in the hospital database, and DOD_SSN refers to the date of death in a social security database. The screenshot to the right of Fig. 9 shows that the values of DOD_HOSP and DOD_SSN are different. From the screenshot to the left of Fig. 9, it can be deduced that DOD is the combination of records of DOD_HOSP and DOD_SS, and DOD_HOSP has a higher priority for adoption if both values exist.

**Fig. 10:**
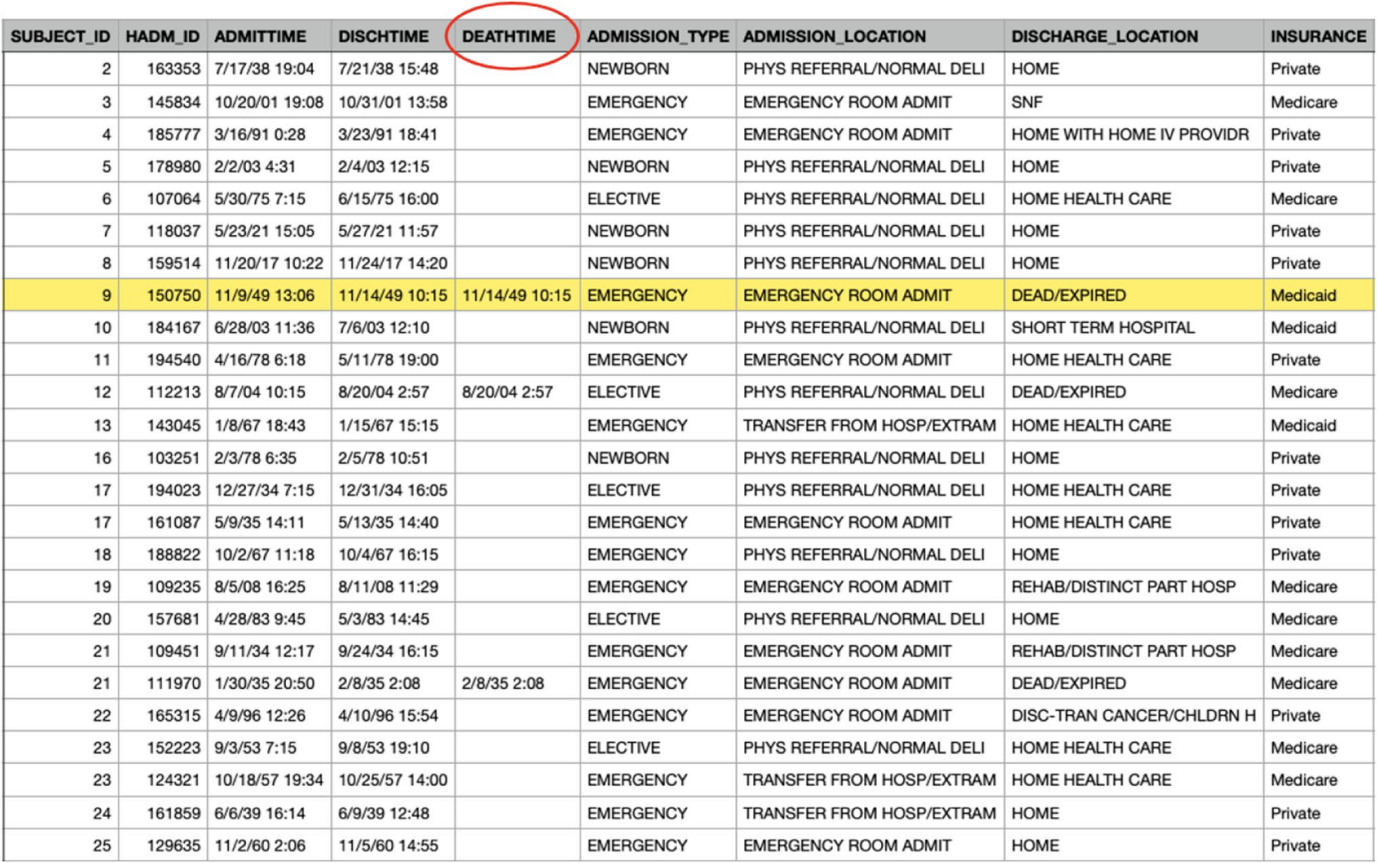
Admission table in MIMIC data sets

There is also a DEATHTIME in the Admission Table (Fig. 10). The comparison demonstrates that records of death times in the two tables are inconsistent; for example, in *Patient* table, the death time of HADM_ID = 9 is 11/14/49 0:00; while in *Admission* table, the record is 11 /14/49 10:15. As the times in all records in the *Patient* table are 0:00, it is assumed that the record in the *Admission* table is more accurate.

Thus, on the basis of the above observations, semantic ambiguity is generated if the data source has not been shown to data users; this leads to misjudgements during data analysis. Semantic ambiguity, a typical type of data quality problem that occurs often, has been identified as the cause of such issues because the FHIR specification cannot enumerate all matching situations for local databases.

The ostensive approach has the capacity to reduce this type of ambiguity by providing the sources of data. The source of DOD_HOSP, DOD_SSN and DEATHTIME can be found by retrieving the *table*, *property,* and *source* attributes from the knowledge graph, as a similar process to a ‘reverse lookup’.

The following query would return all sources and tables by initially searching for all mappings that link to the FHIR Patient attribute ‘deceasedDateTime’.
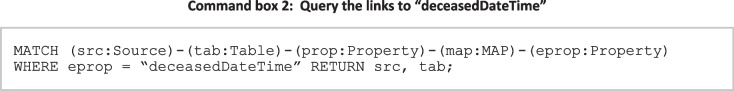


In order to identify the source, a query must be run on that source dataset to identify matching values. Such an operation is for data users to figure out how the FHIR implementer maps local data to FHIR, which is beneficial for the data users to use data correctly. For example, for a datetime x and patient ID y this would be converted into the following queries.
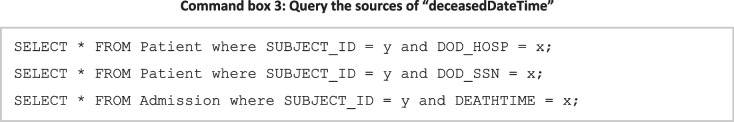


In summary, the Semantic Engine performs the lexical- and ostensive approach through semantic searching facilitated by the FHIR knowledge graph, retrieving data and their context as examples from local clinical systems. Furthermore, specifying primary data sources in local datasets through the construction of mapping relationships can prevent data conflicts in data exchange processes.

### Querying Blood Glucose Levels in the FIHR Defined Format

In this section, an example is used to explain how data can be retrieved from multiple institutional EHRs in FHIR format.

This case study queries blood glucose levels from MIMIC and diabetes datasets in FHIR format by using the Semantic Engine; the query is posed to the system using FHIR terminology. In this instance, all *observations* related to a patient, which are blood glucose measurements, are the object of the research.** Command box 4** details the query in SQL format. The *observation* in FHIR is used to model the result of medical observations, while the *coding* property of FHIR is used to denote the type of test. For this example, it is assumed that LOINC codes (McDonald et al., 2003) are used to code medical observations. Within an observation, ‘subject.reference’ refers to the patient with ID 1.
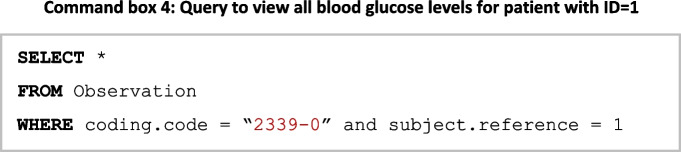


Using the query SQL format, the next step is to query the Semantic Engine in order to determine what sources are required in order to fulfil the query. This is achieved by examining all mapping nodes connected to an FHIR *observation*.

**Fig. 11:**
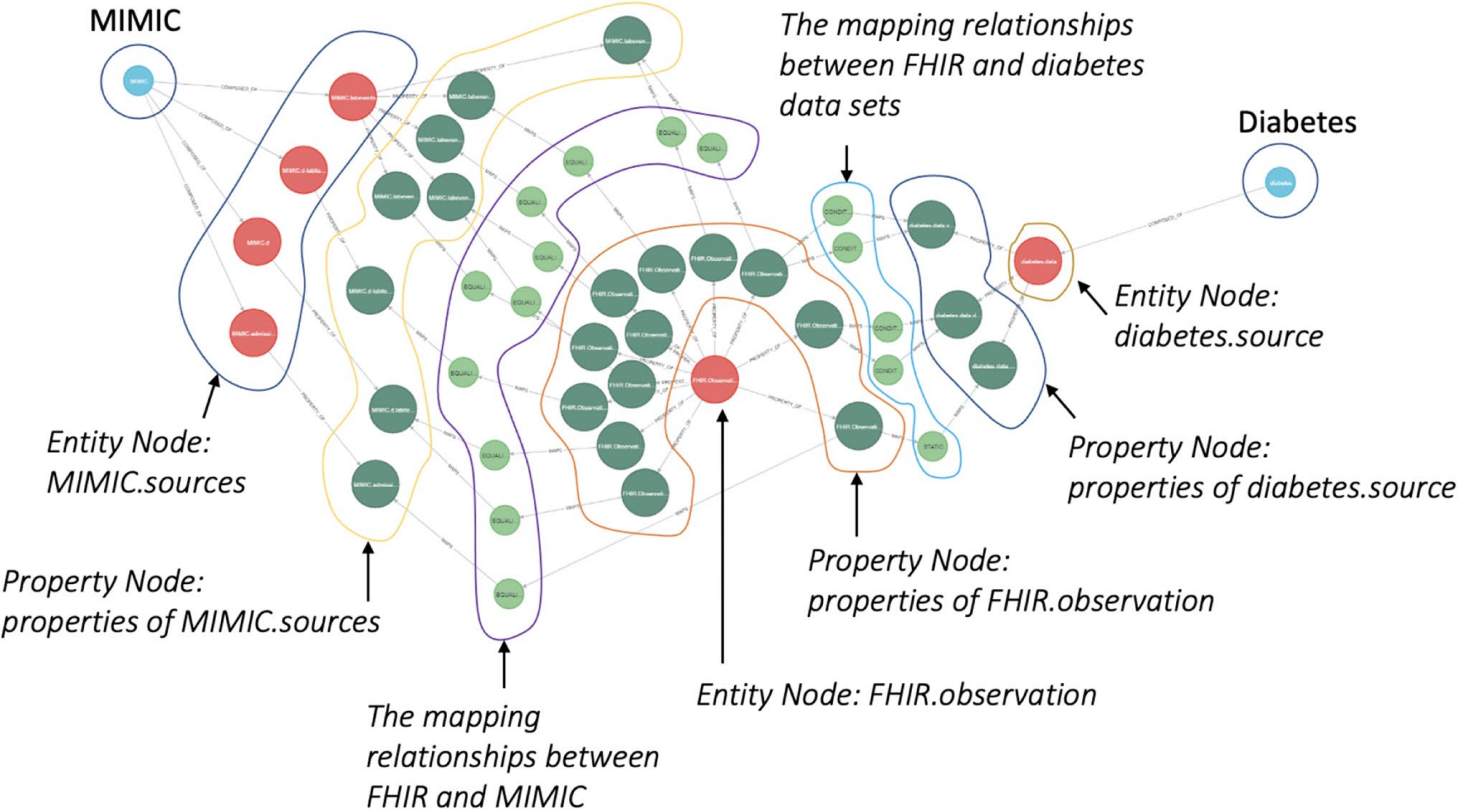
Mappings for FHIR Observation

Figure 11 details the mappings for an FHIR *observation* of both the MIMIC data and the diabetes datasets. The FHIR knowledge graph sits at the centre of the Semantic Engine and remains stable unless FHIR evolves to a new version. The MIMIC and diabetes datasets are connected to the FHIR knowledge graph through schema mapping (Section 5.2.2) and data mapping (Section 5.2.3). When a new data source is connected to the FHIR knowledge graph, the Semantic Engine is updated.

#### Query Processing

After identification of what source(s) are required to fulfil the query, in this case the diabetes data and the MIMIC dataset, the next step is to translate the query into a format that can be read by each mapping connector.

The diabetes data are a single-source dataset; thus, in this instance, manual mappings provided by domain experts are required to match FHIR entities to the dimensions within the diabetes dataset schema. This is achieved by examining the mapping nodes between the diabetes dataset and FHIR shown in Table 2.

**Table 2:**
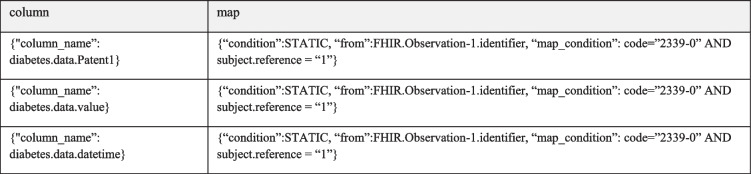
Mappings for the diabetes dataset

From these mappings, it can be observed that the ‘patient’ attribute is a STATIC value embedded within the mappings, the attribute ‘value’ from ‘observation’ maps directly to the column ‘value’ within the diabetes dataset and that the FHIR attribute ‘issued’ maps to the ‘datetime’ attribute within the diabetes dataset. The STATIC value represents an annotation to the source data to supply required semantics in order to achieve integration. In this instance as the source dataset only contains the dimensions datetime and value annotated information such as the Patient is required as a static annotation to the source dataset. An example of the dataset with semantic annotations can be seen in Table 6.

Examining these mappings, which describe the source data, a comparative SQL query to extract the data from their respective source can be seen in **Command box 5**.
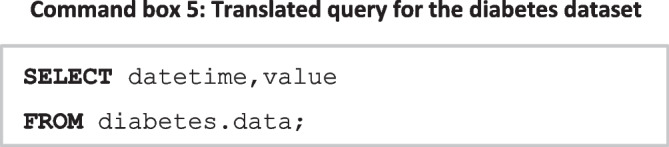


**Fig. 12:**
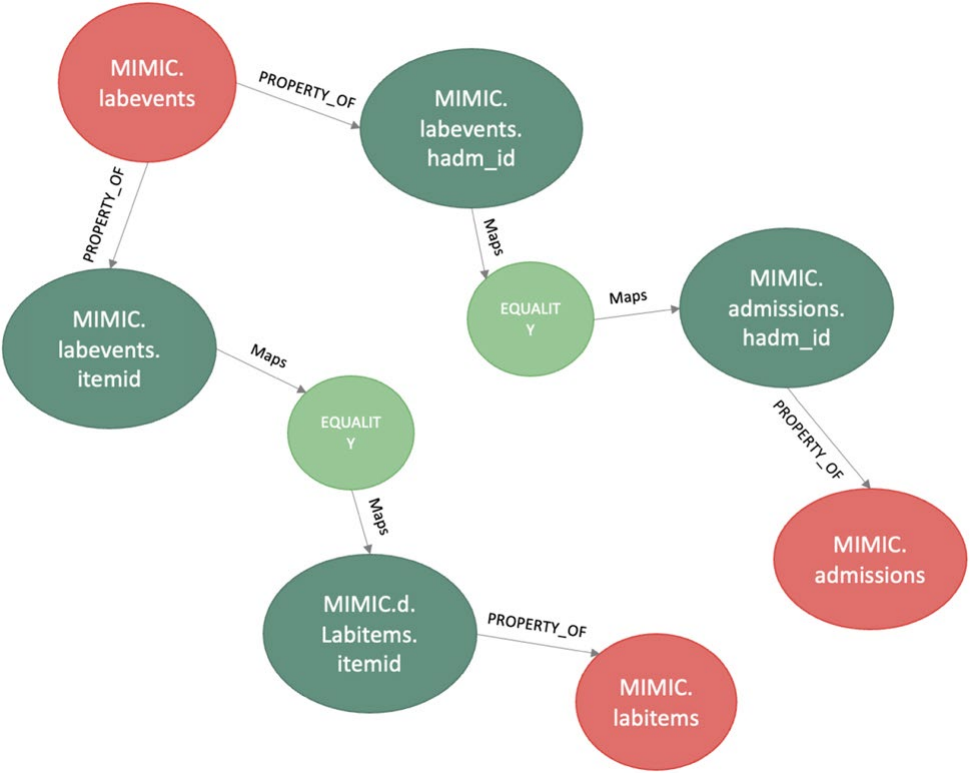
MIMIC internal mappings

The MIMIC dataset shows that an *observation* in FHIR is represented by the tables Lab events, Admissions, Patients, and D_Labitems within MIMIC. A query for these data requires a join across these tables, necessitating knowledge of the MIMIC schema. The knowledge graph can be queried to identify these internal joins to translate the query (Fig. 12); a tabular representation of these joins is presented in Table 3 where *entity* refers to an FHIR entity, and *property* relates to the property of the entity. Each row of the table represents a mapping across entities within FHIR and the properties that join the above-mentioned entities.

**Table 3:** Tabular representation of MIMIC internal mappings presented in Fig. 11

Entity	Property
Labevents	MIMIC.labevents,itemid
Labevents	MIMIC.labevents.hadm_id
Labitems	MIMIC.labitems.itemid
Admissions	MIMIC.admissions.hadm_id

By examining these relationships, the query can be translated into a similar one that can query data in the MIMIC schema (**Command box 6**).
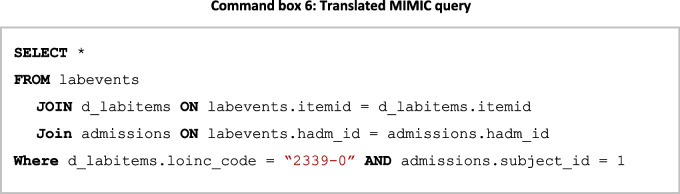


The query is now translated and can be passed to the local clinical database for the retrieval of data according to the mapping relationships.

#### Combining Data from Multiple Data Sources

Each query passed to the local clinical database returns a csv file. The data returned from the MIMIC and diabetes queries are shown in Tables 4 and 5 respectively. The next steps are to convert these files into FHIR format and integrate them in order to return a unified view.

**Table 4:** Data returned from MIMIC

Subject_ID	HADM_ID	ITEMID	CHARTTIME	VALUE	VALUENUM	VALUEUOM	FLAG	LABEL	FLUDID	CATEGORY	LOINC_CODE
1	1	50,809	20/10/2018 20:04	265	265	mg/dL	abnormal	Glucose	Blood	Blood gas	2339–0
1	1	50,809	20/10/2018 21:51	267	267	mg/dL	abnormal	Glucose	Blood	Blood gas	2339–0
1	1	50,809	21/10/2018 00:42	299	299	mg/dL	abnormal	Glucose	Blood	Blood gas	2339–0
1	1	50,809	21/10/2018 01:46	294	294	mg/dL	abnormal	Glucose	Blood	Blood gas	2339–0

**Table 5:** Data returned from diabetes query

DATETIME	VALUE
23/10/2018 08:00	354
23/10/2018 18:00	275

In the case of sparse data sources, these may **not contain** sufficient information to correctly integrate data into FHIR. For example, the diabetes data (Table 5) contains only two columns, i.e., datetime and value. These data require annotation with static semantic data in order to be integrated with FHIR; these semantic annotations are embedded within the mapping nodes with the static identifier. These STATIC mappings are used in conjunction with the mappings derived from the source to facilitate semantic integration within the knowledge graph.

For the diabetes dataset, the static data requiring annotation are patient id, the LOINC code, and the unit of measurement. This produces an intermediate csv file, as shown in Table 6.

**Table 6:** Diabetes data after annotation

DATETIME	VALUE	FHIR.Observation.subject.reference	FHIR.Observation.coding.code	FHIR.Observation.Unit
23/10/2018 08:00	354	1	2339–0	mg/dL
23/10/2018 18:00	275	1	2339–0	mg/dL

The next step of the process is the re-examination of the mappings in order to transform each attribute into FHIR. This is achieved by re-examining the mappings to determine how attributes returned map to FHIR, and by applying any transformations embedded within the mapping nodes. Any attributes that contain no mappings are disregarded. The MIMIC data and diabetes data after this re-mapping are shown in Tables 7 and 8 respectively.

**Table 7:** MIMIC data after re-mapping

FHIR.Observation.subject.reference	FHIR.Observation.issued	FHIR.Observation.value	FHIR.Observation.Unit	FHIR.Observation.coding.code
1	20/10/2018 20:04	265	mg/dL	2339–0
1	20/10/2018 21:51	267	mg/dL	2339–0
1	20/10/2018 00:42	299	mg/dL	2339–0
1	20/10/2018 01:46	294	mg/dL	2339–0

**Table 8:** Diabetes data after re-mapping

FHIR.Observation.issued	FHIR.Observation.value	FHIR.Observation.subject.reference	FHIR.Observation.coding.code	FHIR.Observation.Unit
23/10/2018 08:00	354	1	2339–0	mg/dL
23/10/2018 18:00	275	1	2339–0	mg/dL

Finally, the two datasets require integration. A previous work (Scriney et al., 2019) proposes a methodology for the determination of an integration strategy by examining the common datasets for each source in order to design a common data model. In this study, the common data model is the FHIR JSON schema. Using this methodology, the row-append method is selected to produce the unified data mart shown in Table 9.

**Table 9:** FHIR Observation response of diabetes data

FHIR.Observation.subject.reference	FHIR.Observation.issued	FHIR.Observation.value	FHIR.Observation.Unit	FHIR.Observation.coding.code
1	20/10/2018 20:04	265	mg/dL	2339–0
1	20/10/2018 21:51	267	mg/dL	2339–0
1	20/10/2018 00:42	299	mg/dL	2339–0
1	20/10/2018 01:46	294	mg/dL	2339–0
1	23/10/2018 08:00	354	mg/dL	2339–0
1	23/10/2018 18:00	275	mg/dL	2339–0

## Discussion and Conclusions

Interestingly, during the review process of this paper, FHIR released its latest version (FHIR v4B, released on May 28, 2022), which discusses the issue of conformality. FHIR v4.3.0 introduces a conformance layer (HL7 International, 2022) to mitigate the interoperability problem caused by the inconsistent use of FHIR specifications by different applications, which is the third type of semantic ambiguity discussed in Section 2. The conformance layer is a statement provided by implementers about how the *resources* and their exchange paradigms are used to solve particular use cases, comprising a value set, a structure definition, a capability statement, and an implementation guide. The conformance layer is similar to the *extension* publishing management, which can improve the FHIR conformality, but challenges nevertheless remain.

The proposed ostensive architecture is demonstrated by the prototype of the Semantic Engine to enable data exchange and improve semantic interoperability in this research study. The work has been partially tested in a project supported by the Government of the Republic of Ireland in 2021, which involved multiple data sources for COVID-19 data analytics; a relational database was constructed to interpret semantics, and acts as the Semantic Engine.

This research broadens the scope of the application of FHIR in healthcare ecosystems. The data from heterogeneous sources, such as smart devices, can be interchanged with clinical data via the Semantic Engine.

### The Functionalities of the Semantic Engine

The main functionalities of the Semantic Engine can be summarised in the following four respects:

1. Semantic reasoning

Underpinned by Neo4j, the concepts or resources defined in FHIR are explained through the connected properties of nodes and their relationships. This schematized FHIR data naturally develops the capacity for semantic reasoning between clinical concepts.

The steps for general semantic reasoning are summarised as follows.

- **Data acquisition:**
  A query enters the system in FHIR format. From this query, a list of entities is obtained, represented as **e** and their properties as **e**_**p**_ which are required to deliver the query. Where e_p_ is a subset of all available properties within an entity.Data source (**src**) which can satisfy this query are discovered through a traversal of the knowledge graph identifying mapping nodes (**m**) which link to these properties. Mapping nodes (m) can subsequently be viewed as relations between properties (ep) and sources.

The following query (**Command box 7**) identifies any mappings for a given property (**e**_**p**_) and returns the data source (**src**), the relevant entity (**tab**) and the property required (**prop**).
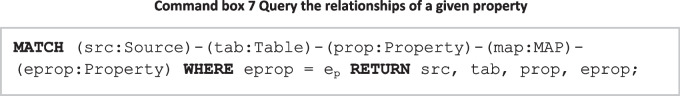


For each mapping, the data source (**src**) is queried in order to return the defined properties (**prop**). The data obtained from the source systems are then converted back into FHIR format (as specified in Section 6.2.2).

2. Patient-centric data organisation

The organisation of data into a patient-centric approach is the premise of achieving patient-centric care. From the perspective of the stakeholders of the healthcare ecosystem, ranging from clinicians to carers, legal practitioners, and taxpayers, a wide range of individuals seek to obtain a holistic view of every individual patient’s case. The benefits and challenges of this have been addressed by academics from many different fields (Pelzang, 2010), with the organisation of patient data used in a patient-centric approach representing the first step towards the elimination of the silos between the health systems.

To facilitate semantic interoperability, the Semantic Engine organises the distributed healthcare data relating to patients and reflects the logic of diagnosis and treatment. Therefore, in addition to the provision of holistic patient health information which can be presented via the Semantic Engine, the patient him/herself can be empowered to authorise which data can be accessed and used by which organisations and agencies. This function can be achieved by the use of an extra module of data authority management, which is not discussed in detail in this paper. Moreover, patients may not be aware of the consequences of their own choices, which is an issue worthy of further exploration from the perspective of healthcare management.

3. Enhancing semantic interoperability

In addition to the definition and interpretation of medical terms, the Semantic Engine can retrieve data from disparate local databases to further clarify the meaning of definitions by providing examples. Through this ostensive approach, the ambiguity caused by lexical definition can be minimised.

In the process of designing the verification scenarios, this study identifies a problem with unclear data sources, which potentially poses challenges for subsequent data analysis processes. The same FHIR *resource*, *observation*, has been used to interpret data collected from patient-worn monitoring equipment and clinical equipment in hospital settings. On consideration of the level of data reliability needed to support patient-centred diagnosis, it is clear that patient-worn monitoring equipment is less reliable than clinical equipment used in a clinical setting. Therefore, in practice, physicians should carefully review the laboratory reports and only use the data provided by the monitoring equipment for reference. In order to provide a firm foundation for an information-assisted clinical diagnosis system, the limitations of this study and suggestions for future research are discussed.

4. Applicability in other fields

This proposed information architecture processes semantics and data separately to avoid privacy and security issues arising from centralised data storage, while support information can be exchanged across heterogeneous databases. This architecture can be applied in other domains that require information exchange and communication between dispersed systems. The construction of a consensus knowledge graph is the premise of the application of this semantic-data separated architecture.

### The Limitations of the Research Study

This study adopts FHIR as domain knowledge to construct a Semantic Engine for the interpretation of the meanings of clinical concepts. It is observed that FHIR does not distinguish between the different levels of data reliability. When this ostensive information architecture is brought into use, consideration should be given to the level of data reliability and the conflicts caused by multiple data sources being used for the same indicator. This problem can potentially be solved by specifying the primary database, although due to the limited availability of medical data, this study does not provide an in-depth discussion of this issue.

This study focuses on semantic interoperability but does not explore the relationship between semantics and operational processes, such as patient pathways or clinical procedures. The context of the data is an extremely important factor concerning their semantics and may vary in the different processes, which this study does not explore in depth.

The ostensive information architecture proposed by this study is applicable to the entire medical ecosystem, therefore, it is evident that there is a serious problem of record linkage, specifically in terms of detecting, identifying, matching, and merging records across heterogeneous databases that relate to the same patient (Reyes-Galaviz et al., 2017). For example, two systems may refer to the same patient but use different identity codes to correctly identify a patient across systems. To overcome these issues, this study proposes a model of **record linker** in the transformation layer; this is similar to the method proposed by Nie and Roantree (Nie & Roantree, 2019) which seeks to produce a probabilistic means of identifying patients during the re-mapping process. Due to the fact that no public medical data contains patient details in order to protect patient privacy, the difficulty of obtaining data on patients’ profiles from multiple systems precludes the conduct of a case study in this study to demonstrate how the record linker works.

### Implications for Future Research

This proposal of the ostensive information architecture simply represents the first step towards the achievement of patient-centric diagnosis. The following are directions worthy of further exploration:

- In the current Semantic Engine, the properties for edges are limited, which indicates the affiliation between nodes. In further research, richer semantic properties could be added to the edges. For example, data from a wearable device could be given low priority if there are data for the exact measurement from a medical device. The richer semantic properties can support the Semantic Engine in the construction of a diagnostic graph, which has the capacity to reason and prioritise the level of data reliability according to its sources.
- Breakthroughs in the medical field and the discovery of new diseases mean that the definitions of clinical concepts are in constant evolution; this underlines the fact that a gap between the Semantic Engine and the data examples is likely to persist. Therefore, the function of tracing and managing the changes of FHIR resources becomes essential to ensuring rigour in the mapping of relationships. Blockchain technology offers an optional solution to this challenge; it can be used to record the evolutionary history of FHIR whilst also tracking the changes in patients’ medical history records (Zhang et al., 2018). The use of blockchain technology in healthcare information systems has many potential application scenarios and is of high practical value (Mettler, 2016). For example, blockchain can be used to provide access to medical data (Azaria et al., 2016) and privacy control (Yue et al., 2016). Overall, the proposed ostensive information architecture provides a foundation for HISs; additional research work, including mapping of organisations, patient pathways, and clinical processes to the Semantic Engine, should be based on a comprehensive HIS proposal.

### Conclusion

In this study, in order to enhance the semantic interoperability of FHIR, and also consider the data privacy issues and regulatory requirements for data sharing, an ostensive information architecture is proposed that separates semantic processing from clinical data storage. There is deliberate separation of semantic schema and underlying data, with the aim of improving flexibility and scalability. The centralised FHIR knowledge graph has the capacity to reduce the cost of the application of FHIR to multiple disparate clinical systems, and is also be flexible in its evolution. This study summarises the benefits of the semantics-data separated architecture into three principal points, as follows:

1. The centralised deployment of FHIR can reduce the costs incurred by its separate deployment in individual local systems, alleviating the impact of its evolution. Our system is a federated architecture where queries are first to run on their respective sources and the data returned are mapped and integrated using the Semantic Engine, returning a unified view of the data. As we do not envisage incremental updates to the Semantic Engine, it is possible to alleviate potential time costs within the integration and mapping steps by hosting multiple instances of the FHIR knowledge graph within the cloud.
2. This architecture acquires horizontal scalability through the maintenance of the distributed storage of clinical data and the deployment of the centralised FHIR knowledge graph layer in the cloud cluster. This architecture supports vertical scalability in terms of handling complex semantic reasoning and the evolution of FHIR.
3. The abstract semantic layer provides patients with the capacity to gain a complete view of their healthcare from dispersed data sources, enabling them to precisely decide the degree and extent of information exposure by managing the access permissions that can be embedded in the Semantic Engine. The Semantic Engine executes the role-based accessed management tasks without exposing the FHIR knowledge graph to patients.

## Data Availability

The data that support the findings of this study are openly available in zenodo at 10.5281/zenodo.7545834.
